# Supplementary material for: Gut microbiota and fermentation-derived branched chain hydroxy acids mediate health benefits of yogurt consumption in obese mice
Source: Nat Commun. 2022 Mar 15;13:1343. doi: 10.1038/s41467-022-29005-0 (PMC8924213; doi:10.1038/s41467-022-29005-0)
Supplement: Supplementary file 1 — Supplementary Information [file 41467_2022_29005_MOESM1_ESM.pdf]

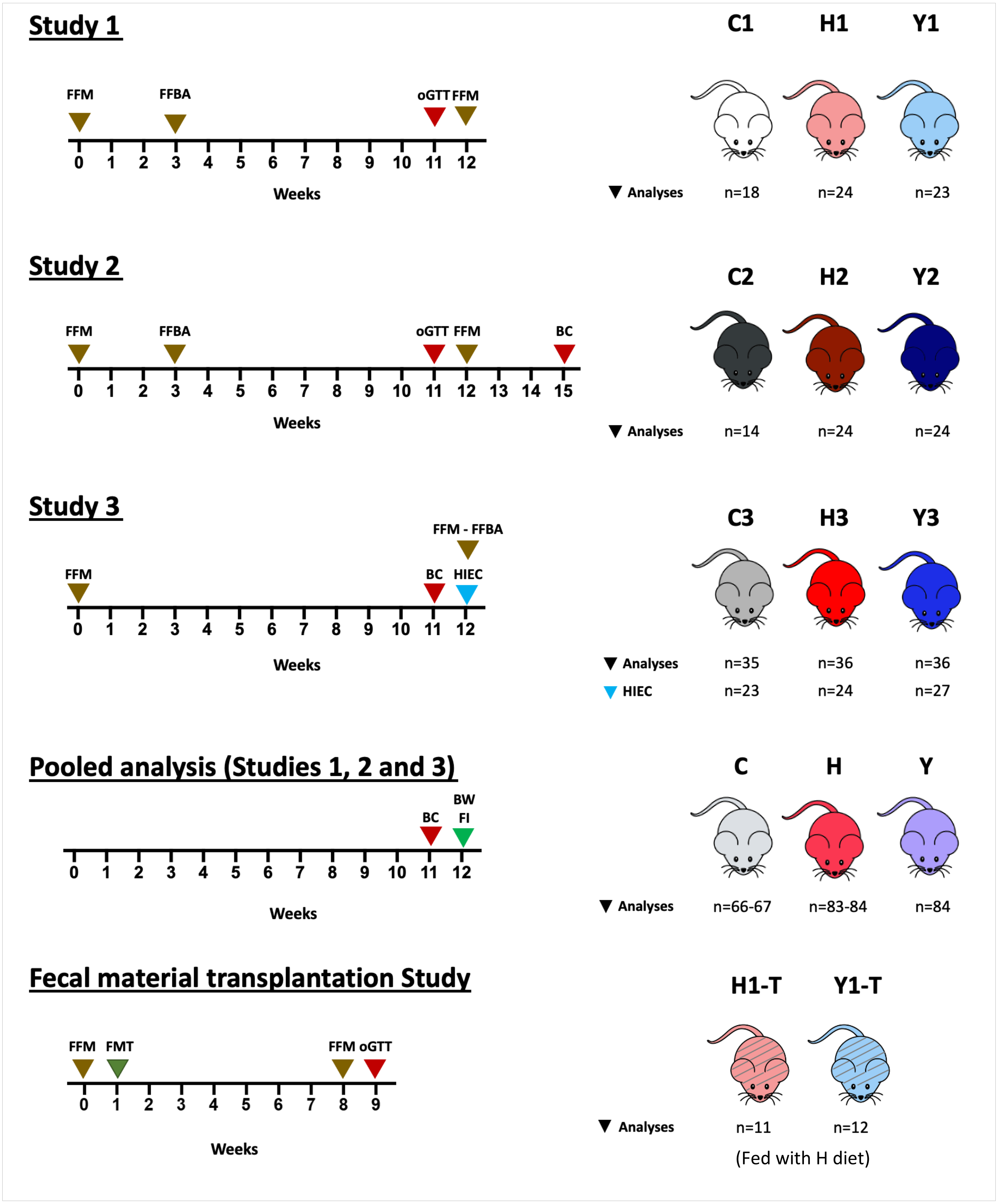

Supplementary Figure 1. Experimental study design. Four different cohorts of mice were used for the purpose of the project. The Study 1 and Study 2 were carried out to explore the overall impact of yogurt intake on metabolic homeostasis. The Study 3 was designed to investigate the whole body, hepatic and peripheral insulin sensitivity using the HIEC technique. Number of mice for the HIEC was lower because it only included those mice that were successfully catheterized. The last study is germ-free animals that underwent a fecal material transplantation used germ-free animals to determine the causal role of the gut microbiota in yogurt-related beneficial effects. Study 1: n=18-24; Study 2: n=14-24; Study 3: n=23-27 or n=35-36 mice depending on the performed analyses and Germ-free study: n=11-12 biologically independent mice. BC: Blood collection; BW: Body Weight; C: low-fat low-sucrose control diet; FFBA: Fresh feces collection for Bile Acid analysis; FFM: Fresh Feces collection for Microbiota analysis; FI: Food Intake; oGTT: oral Glucose Tolerance Test; H: high-fat high-sucrose diet with a protein mixture replacing casein; HIEC: Hyper-insulinemic-Euglycemic Clamp; H1-T: germ-free mice transplanted with feces from H1 mice; Y: lyophilized yogurt incorporated in H diet; Y1-T: germ-free mice transplanted with feces from Y1 mice. Numbers (1, 2, 3) refer to the study affiliation.

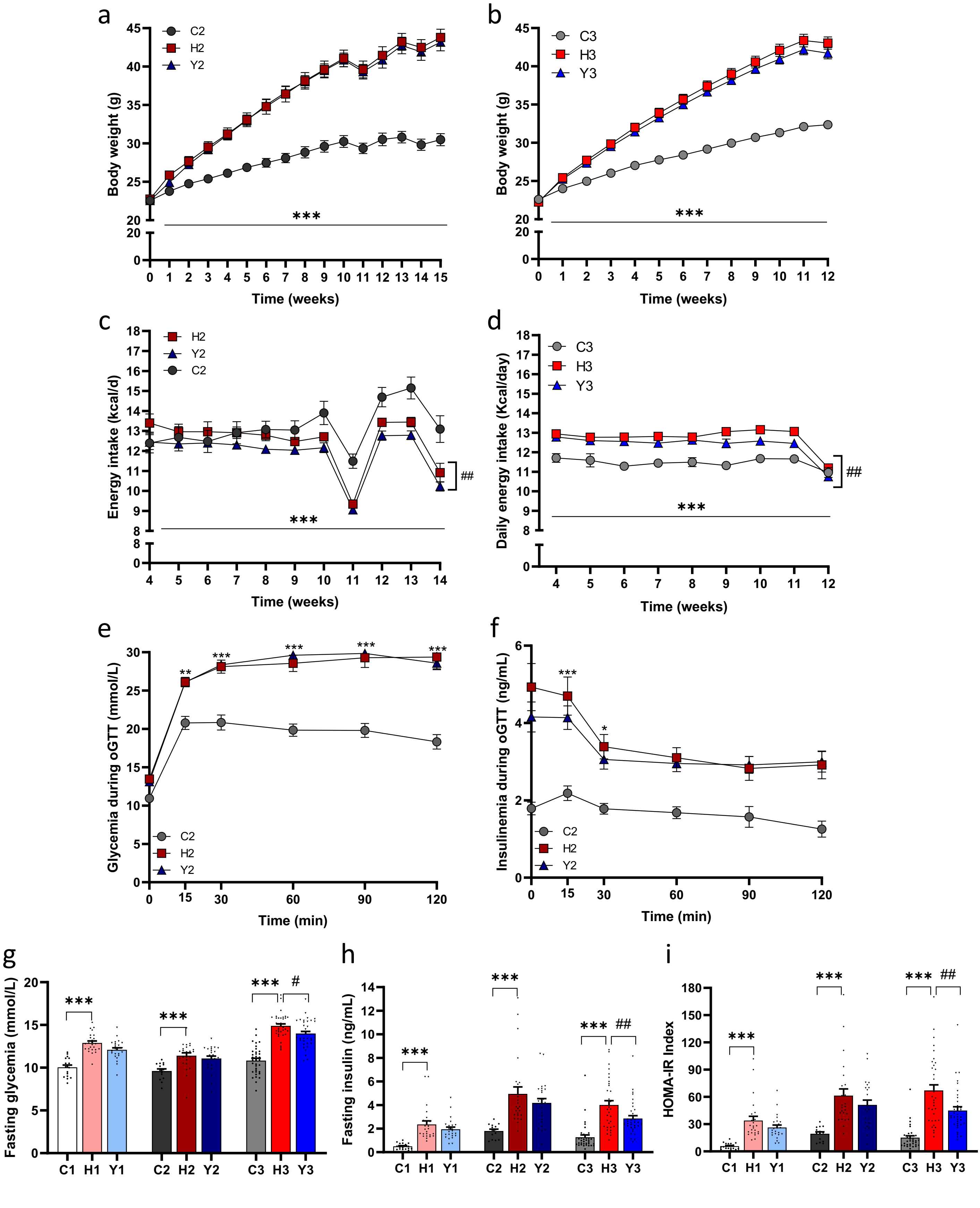

Supplementary Figure 2. Body weight, energy intake record and glucose homeostasis in individual Studies. (a and b) Body weight throughout the Study 2 and 3. (c and d) Energy intake recorded from week 4 to week 12 throughout the Study 2 and 3. (e) Glucose tolerance test at week 11 in Study 2. (f) Insulin response following the oral glucose challenge in Study 2. (g-i) Detailed fasting (g) glucose, (h) insulin and corresponding (i) HOMA-IR index of the three Studies after 12 weeks of treatment. Study 1: n=17-24; Study 2: n=14-24 and Study 3: n=35 biologically independent mice. Data are expressed as mean ± SEM. H versus C: ***$p < 0.001$. Y versus H: $^{\#}p < 0.05$, $^{\#\#}p < 0.01$. C: low-fat low-sucrose control diet; H: high-fat high-sucrose diet with a protein mixture replacing casein; Y: lyophilized yogurt incorporated in H diet. Numbers (1, 2, 3) refer to the study affiliation. Two-way repeated measures anova with (a-b,e-f) or without (c-d) baseline adjustment. ANOVAs or Mann-Whitney tests depending on data distributions (g-i). All tests were two-sided.

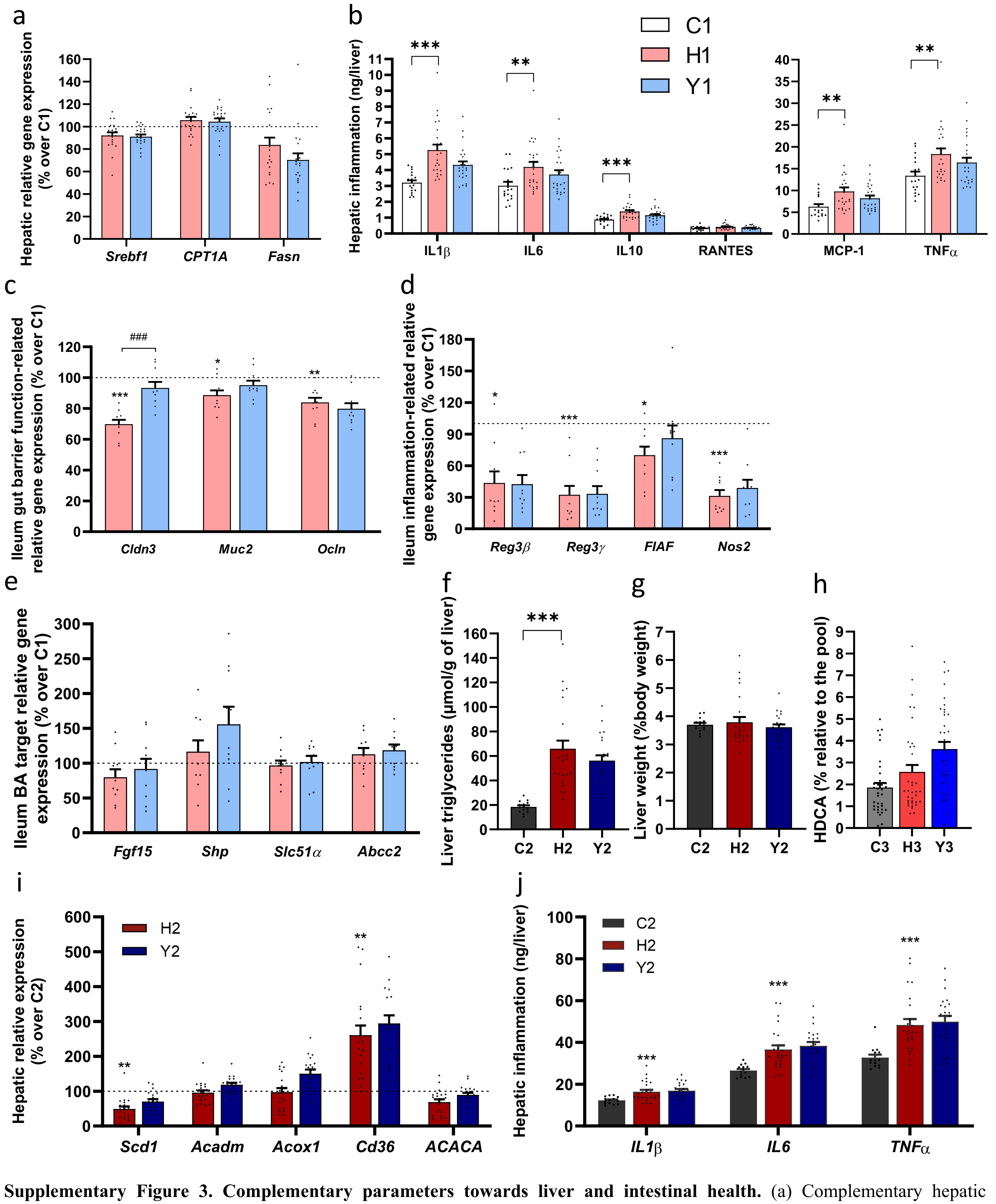

Supplementary Figure 3. Complementary parameters towards liver and intestinal health. (a) Complementary hepatic relative gene expression in Study 1. (b) Hepatic inflammation in Study 1 analyzed by a Multiplex assay. (c-e) Ileum (c) gut barrier function-related gene expression, (d) inflammation-related gene expression and (e) bile acid (BA) target gene expression in Study 1. (f) Liver weight and (g) triglyceride content in Study 2. (f) Fecal hyodeoxycholic acid content measured at week 12 in Study 3. (i) Hepatic relative gene expression and (j) inflammation in Study 2 analyzed by ELISA kit. Study 1: n=18-24 except for panel (a) (n=8 for C1 group and n=20 for H1 and Y1 groups) and (c-e) (n=7-10); Study 2: n=14-24 and Study 3: n=35 biologically independent mice. Data are expressed as mean ± SEM. H versus C: *$p < 0.05$, **$p < 0.01$, ***$p < 0.001$. Y versus H: ###$p < 0.001$. For panels c and e-g (data expressed in % over C), C group is considered as a reference and represented by a dash line. C: low-fat low-sucrose control diet; H: high-fat high sucrose diet with a protein mixture replacing casein; Y: lyophilized yogurt incorporated in H diet. Numbers (1, 2, 3) refer to the study affiliation. One-way ANOVAs or Mann-Whitney tests depending on data distribution (a-h). Benjamini-Hochberg adjustment for multiple testing within each experiment and each type of variable (e.g. study 1 - liver qPCR, study 1 - liver cytokines) (a-b, i-j). All tests were two-sided.

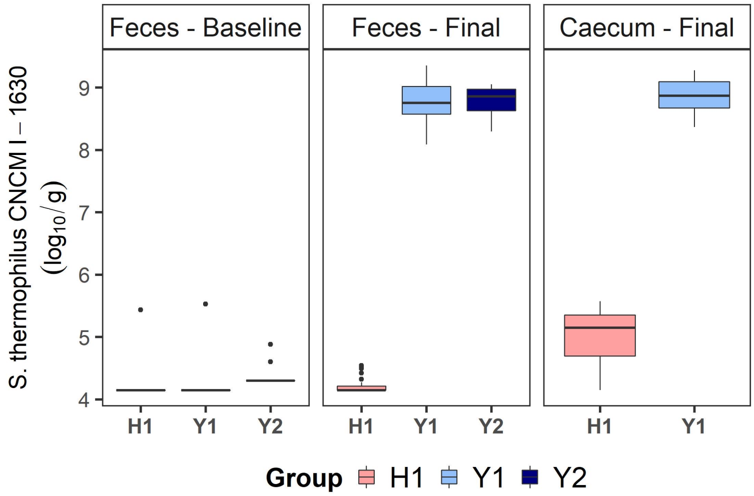

Supplementary Figure 4. ***S. thermophilus*** **CNCM I-1630 quantity in fecal and caecal contents.** Boxplots of qPCR results for strain *S. thermophilus* CNCM I-1630 in feces and caecum (respectively n=24 Study 1 feces, n=21 Study 2 feces and n=12 Study 1 caecum). The thick black line is the median, the box spans from Q1 (25th percentile) to Q3 (75th percentile) and the whiskers extend to the most extreme observation within 1.5 times the interquartile range (Q3-Q1) from the nearest quartile, so that outliers >1.5 times the interquartile range are individually displayed. Data are expressed as log_{10}/g of feces or caecum content. Data below the lower limit of quantification were imputed to the limit of quantification (4.2 log_{10}/g for Study 1 and 4.3 log_{10}/g for Study 2). C: low-fat low-sucrose control diet; H: high-fat high sucrose diet with a protein mixture replacing casein; Y: lyophilized yogurt incorporated in H diet. Numbers (1, 2) refer to the study affiliation.

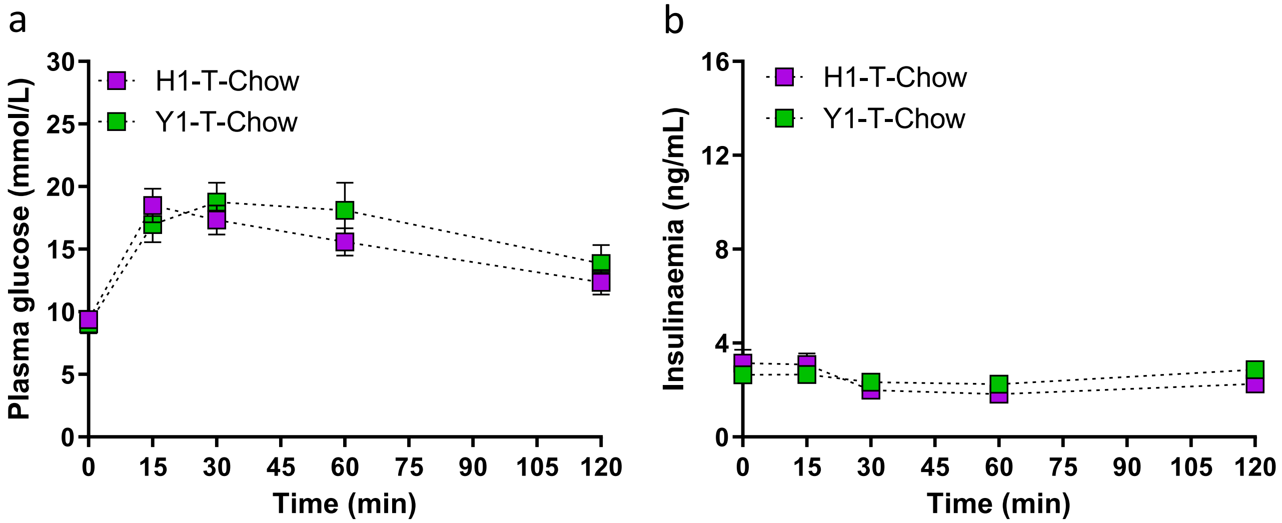

Supplementary Figure 5. Glucose tolerance test (GTT) of gnotobiotic mice fed with chow diet. (a) glycemic and (b) insulinemic responses of germ-free mice transplanted with feces from H1-fed mice and Y1-fed mice, during the GTT. Mice were issued from the same cohort than mice used in the current study (main paper) but were fed chow diet and not high-fat. They were fasted 4h before the GTT. Data are expressed as mean ± SEM. Two-way ANOVA with repeated measures followed by Tukey post-hoc test. H1-T: n=11 and Y1-T: n=8 biologically independent mice.

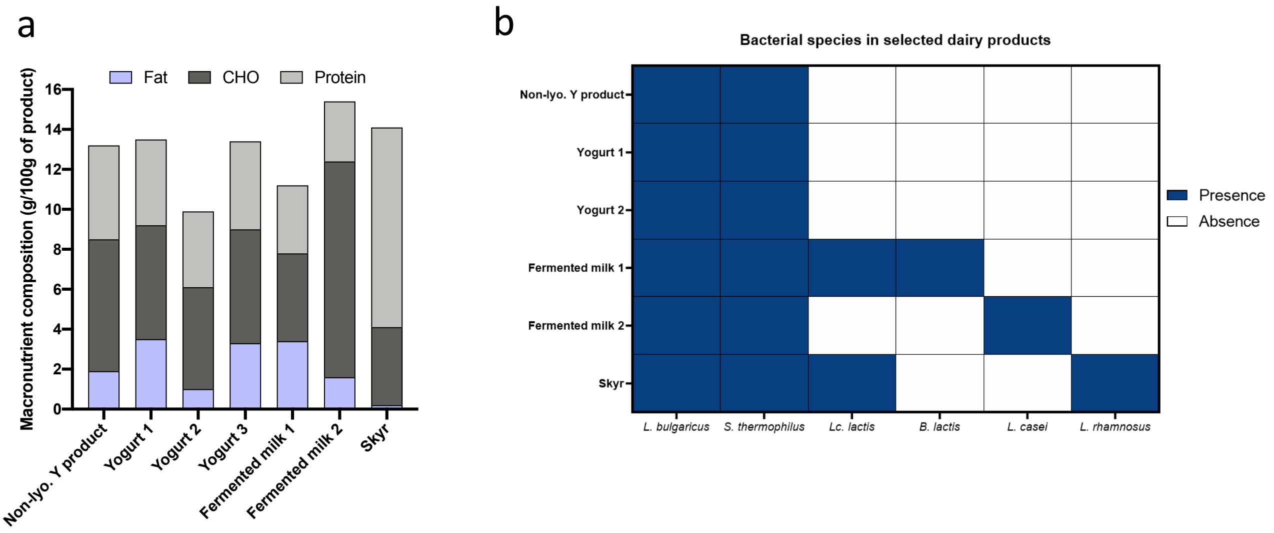

Supplementary Figure 6. Composition of the various selected dairy products. (a) Macronutrient composition and (b) presence of bacterial species used for fermentation. B: *Bifidobacterium*; CHO: carbohydrates; L: *Lactobacillus*; Lc: *Lactococcus*; S: *Streptococcus*.

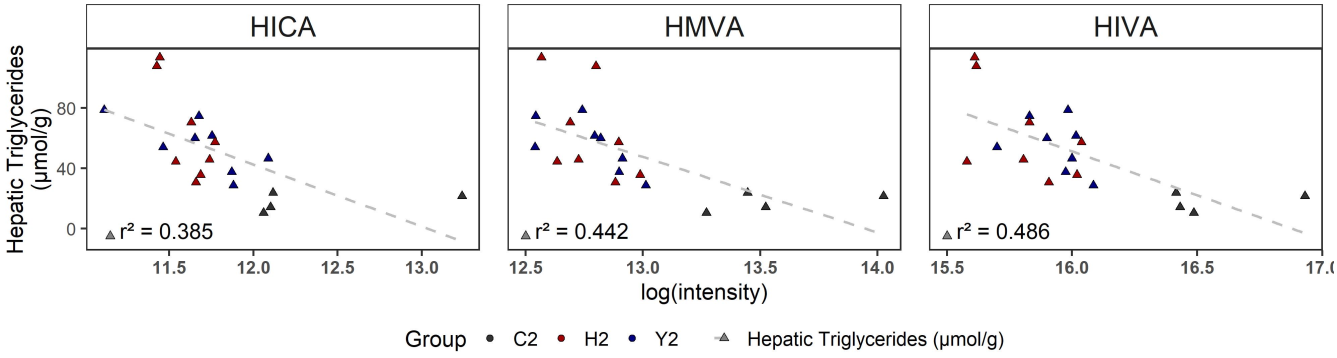

	p-value	Adj. p-value	p-value	Adj. p-value	p-value	Adj. p-value
**Liver TG**	**0.004**	**0.004**	**0.001**	**0.002**	**<0.001**	**0.002**

Supplemental Figure 7. Hepatic branched chain hydroxy acids (BCHA) correlate with hepatic triglycerides. Pearson correlations between hepatic each BCHA levels and hepatic triglycerides in Study 2. Benjamini-Hochberg correction for multiple testing within each BCHA over all tested parameters (hepatic triglycerides, fasting glucose and fasting insulin). All tests were two-sided. BCHA data were log transformed. n=4-8 biologically independent mice. P-value and adjusted p-value are indicated under each corresponding graph.
