# Supplementary material for: Gut microbiota and fermentation-derived branched chain hydroxy acids mediate health benefits of yogurt consumption in obese mice
Source: Nat Commun. 2022 Mar 15;13:1343. doi: 10.1038/s41467-022-29005-0 (PMC8924213; doi:10.1038/s41467-022-29005-0)
Supplement: Supplementary file 3 — Description of Additional Supplementary Files [file 41467_2022_29005_MOESM3_ESM.docx]

**Description of Additional Supplementary Files**

**Supplementary Data 1**

Description: Supplementary Table 1 - Summary table of parameters analyzed in Studies 1, 2 and 3. Parametric or non-parametric tests depending on data distributions. Parametric: one-way (one time point) or two-way (several time points) ANOVAs. Non-parametric: Mann-Whitney tests.

**Supplementary Data 2**

Description: Supplementary Table 2 - CLP lipidomic profile of liver in Studies 1 and 2. All lipids were grouped according to super pathway. Data are presented as fold of change. Significant (q≤0.05) elevations are indicated by red shading, while significant reductions are colored in green. Trending (0.05<q<0.10) elevations are indicated by light red shading, while trending reductions are colored light green. Lipids with changes consistent with metabolic improvements in Y fed mice are shown in bold. DAG, Diacylglycerol; FFA, Free fatty acid; HCER, Hexosylceramide; LCER, Lactosylceramide; LPC, Lysophosphatidylcholine; LPE, Lysophosphatidylethanolamine; PE, Phosphatidylethanolamine.

**Supplementary Data 3**

Description: Supplementary Table 3 - Global HD4 metabolomic profile of lyophilized milk and yogurt products. All named and unnamed metabolites were grouped according to super pathway. Significant (q≤0.05) elevations are indicated by red shading, while significant reductions are colored in green. Trending (0.05<q<0.10) elevations are indicated by light red shading, while trending reductions are colored light green. The intensity of color indicates above 10-fold differences.

**Supplementary Data 4**

Description: Supplementary Table 4 - Global HD4 metabolomic profile of liver in Studies 1 and 2. All named and unnamed metabolites were grouped according to super pathway. Significant (q≤0.05) elevations are indicated by red shading, while significant reductions are colored in green. Significant (p≤0.05) elevations are indicated by light red shading, while significant reductions are colored in light blue. Two-way ANOVAs. Multiple testing correction by FDR (q-value), comparison-wise. All tests were two-sided.

**Supplementary Data 5**

Description: Supplementary Table 5 - TaqMan® assays for liver gene expression.

**Supplementary Data 6**

Description: Supplementary Table 6 - TaqMan® assays for ileum gene expression.

**Supplementary Data 7**

Description: Supplementary Table 7 - CLP internal standards.

**Supplementary Data 8**

Description: Supplementary Table 8 - CLP methodology.
