# Supplementary material for: Gut microbiota and fermentation-derived branched chain hydroxy acids mediate health benefits of yogurt consumption in obese mice
Source: Nat Commun. 2022 Mar 15;13:1343. doi: 10.1038/s41467-022-29005-0 (PMC8924213; doi:10.1038/s41467-022-29005-0)
Supplement: Supplementary file 12 — Reporting Summary [file 41467_2022_29005_MOESM12_ESM.pdf]

nature portfolio

Corresponding author(s): *Double-blind peer review submissions: write DBPR and your manuscript number here instead of author names.*

Last updated by author(s): *YYYY-MM-DD*

Reporting Summary

Nature Portfolio wishes to improve the reproducibility of the work that we publish. This form provides structure for consistency and transparency in reporting. For further information on Nature Portfolio policies, see our Editorial Policies and the Editorial Policy Checklist.

Statistics

For all statistical analyses, confirm that the following items are present in the figure legend, table legend, main text, or Methods section.

n/a	Confirmed	
☐	☒	The exact sample size (*n*) for each experimental group/condition, given as a discrete number and unit of measurement
☐	☒	A statement on whether measurements were taken from distinct samples or whether the same sample was measured repeatedly
☐	☒	The statistical test(s) used AND whether they are one- or two-sided<br>*Only common tests should be described solely by name; describe more complex techniques in the Methods section.*
☐	☒	A description of all covariates tested
☐	☒	A description of any assumptions or corrections, such as tests of normality and adjustment for multiple comparisons
☐	☒	A full description of the statistical parameters including central tendency (e.g. means) or other basic estimates (e.g. regression coefficient) AND variation (e.g. standard deviation) or associated estimates of uncertainty (e.g. confidence intervals)
☐	☒	For null hypothesis testing, the test statistic (e.g. *F*, *t*, *r*) with confidence intervals, effect sizes, degrees of freedom and *P* value noted<br>*Give P values as exact values whenever suitable.*
☒	☐	For Bayesian analysis, information on the choice of priors and Markov chain Monte Carlo settings
☒	☐	For hierarchical and complex designs, identification of the appropriate level for tests and full reporting of outcomes
☐	☒	Estimates of effect sizes (e.g. Cohen's *d*, Pearson's *r*), indicating how they were calculated

Our web collection on statistics for biologists contains articles on many of the points above.

Software and code

Policy information about availability of computer code

Data collection	Microsoft Excel
Data analysis	Software Prism GraphPad version 8.0 , SAS 9.4, R (several versions from 3.4.0 to 4.0.3), ArrayStudio

For manuscripts utilizing custom algorithms or software that are central to the research but not yet described in published literature, software must be made available to editors and reviewers. We strongly encourage code deposition in a community repository (e.g. GitHub). See the Nature Portfolio guidelines for submitting code & software for further information.

Data

Policy information about availability of data

All manuscripts must include a data availability statement. This statement should provide the following information, where applicable:

- Accession codes, unique identifiers, or web links for publicly available datasets
- A description of any restrictions on data availability
- For clinical datasets or third party data, please ensure that the statement adheres to our policy

The data that support the findings of this study are available from the corresponding author upon reasonable request; the raw data of 16S rRNA sequence for the project through the European Nucleotide Archive (https://www.ebi.ac.uk/ena/) under accession number PRJEB47834 and the raw metabolomics data from MetaboLights (https://www.ebi.ac.uk/metabolights/) under study number MTBLS442.

Field-specific reporting

Please select the one below that is the best fit for your research. If you are not sure, read the appropriate sections before making your selection.

- [x] Life sciences
- [ ] Behavioural & social sciences
- [ ] Ecological, evolutionary & environmental sciences

For a reference copy of the document with all sections, see nature.com/documents/nr-reporting-summary-flat.pdf

Life sciences study design

All studies must disclose on these points even when the disclosure is negative.

Sample size

- Number of animals used in Study 1:

The sample size to validate the model (ie. to induce the glucose intolerance and metabolic endotoxemia) when comparing the C (low fat low sucrose control diet) group vs. the H (high fat high sucrose diet) group was based on data published by Daniel et al., 2021 (and already available in the group at the time of the experiments). In order to induce a statistical difference, as observed between Low-Fat and High-Fat diets from the previous study, the following have been calculated:

• Glucose intolerance expressed as 30% AUC change upon OGTT:
N = 22 of mice per group were needed at a power of 0.95
N = 14 of mice per group were needed at a power of 0.80

• Intestinal inflammation/permeability, expressed as 32% of plasmatic LPS change:
N = 28 of mice per group were needed at a power of 0.95
N =18 of mice per group were needed at a power of 0.80

Based on the above n=18 mice per study group was sufficient to validate the study model with a power of 0.80.

In the absence of preliminary data a 33% margin was used for the sample size to detect the yogurt effect based on the comparison between the H group and the Y (high fat high sucrose diet and lyophylized yogurt product) group.

So n=18 was used for the C group, and n=24 was sued for the H and Y groups.

- Number of animals used in Study 2:

The power calculation to confirm the lyophilized yogurt effect was based on two study groups (group H and Y) using data from Study 1. The primary outcomes were 6h fasting plasma glucose after 15 weeks of lyophilized yogurt intervention and/or insulin level at 15 minutes post glucose bolus during the oral glucose tolerance test after 11 weeks of lyophilized yogurt intervention analyzed as variations vs. baseline.

In first intention, power calculations were performed with the same sample sizes as Study 1 (n=24 for the H and Y groups) except for the C group (n=10).

The following have been calculated:
- For insulin, the power was 81% for the H vs C comparison and 98% for the Y vs H comparison.
- For glucose, the power was above 99% for the H vs C comparison, but only 29% for the Y vs H comparison. To reach 80% power for this last comparison, the number of mice per group would need to be 91. This was, however, not feasible technically.

We thus kept n=24 for the H and Y groups, but increased the C group to n=14, in order to ensure the results for insulin model validation.

- Number of animals used in Study 3:

The primary outcome is the mean difference of glucose infusion rate (GIR) after 12 weeks of diet challenge. The H diet (50% fat, 30% sucrose) represents a newly developed diet for which no prior data on GIR is available. Therefore, literature on challenges with high fat diets of a similar content and duration was used as a basis for power calculation:

55% fat, 7% sucrose diet challenge for 24 weeks
Laplante et al., 2013 reported the hyperinsulinemic-isoglycemic clamp data for C57B1/6J male mice fed ad libitum standard chow diet (SD; Harlan Teklad T-2018) and high-fat diet (HFD, 55% kcal from fat; Harlan Teklad TD-93075) starting at 6 week of age for 24 weeks.

55% fat, 7% sucrose diet challenge for 8 weeks
Xu et al., 2012 reported the hyperinsulinemic-euglycemic clamp data for wild-type C57Bl/6 male mice (Ptpn6f/f : genetic control allele) fed ad libitum standard chow diet (SD) and high-fat diet (HFD, 55% kcal from fat; Harlan Teklad TD-93075) starting at 2 months of age for 8 weeks.

We assessed the power for both effects available on the literature.

The power calculations were estimated for a number of 30 mice per group to detect a difference in mean (H vs. C) using a Student t-test two-sided at a level alpha of 0.05 assuming a GIR of:
- Estimation 1 (Laplante et al., 2013): 16 (sd: 3.8) in H group and 58 (sd: 3.8) in C group ; power equals to > 0.99.
- Estimation 2 (Xu et al., 2012): 10 (sd: 1.0) in H group and 25 (sd: 2.0) in C group ; power equals to > 0.99. Note: an assumption of equal variances between groups was set with sd = 1.5.

The effect of Y associated to the H diet on GIR mean is assumed to be reduced by 20% compared to the H diet, using 30 mice per group the power to detect a difference in mean (H vs. Y) using a Student t-test two-sided at a level alpha of 0.05 assuming a GIR of:
- Estimation 1 (Laplante et al., 2013): 16 (sd: 3.8) in H group and 19.2 (sd: 3.8) in Y group; power equals to 0.89.
- Estimation 2 (Xu et al., 2012): 10 (sd: 1.0) in H group and 12 (sd: 2.0) in Y group; power equals to >0.99. Note: an assumption of equal variances between groups was set with sd = 1.5.
If the effect size is in between the ones observed in Laplante et al., 2013 and Xu et al., 2012, with n=30 mice per group we will able to detect a 20% change in GIR mean with a power of at least 89%.
If the effect size is in between the ones observed in Laplante et al., 2013 (Δ=μ/sd=0.84) and Xu et al., 2012 (Δ=μ/sd=1.33), with n=30 mice per group we will able to detect a change in GIR mean with a power of 80 % between 11.1% to 17,5 %.

There is an anticipated dropout rate of 15% during and following the surgery or during clamp procedure (catheter clothing). Therefore, n=36 of mice per group were placed on different diets in order to reach n=30 for clamp procedure.

- Number of animals used in Germ-free Fecal Material Transplantation Study:
The number of animals for this experiment was determined based on previous projects conducted in our laboratory and power calculation performed for Studies 1, 2 and 3.

- Number of experiments for cell culture assessments:
The number of independent cell experiments was determined based on previous projects conducted in our laboratory.

Data exclusions

Study 1:

After discrepancy verification, we decided to exclude the following mouse values from data analysis:
- Body weight (BW) for mouse H9 at week 12 due a large incoherency in between BW at sacrifice, qNMR test and BW value, possibly due to a clerical error.
- Fasting glucose for mouse C7 at week 12 because the mouse was injured in the neck.
- Glucose value during ITT for mouse Y4 due of aberrant ITT curve.
- Fasting glucose during OGTT for mouse Y5 since this value was abnormally low (below the LFLSD fasting glucose) and could be due to an extended fasting /error to provide the food.
- Glucose value during OGTT of H14 mice due a likely error in glucose gavage (lack of glucose bolus).
- Data of mouse H15 were excluded for kinetics of C-peptide during OGTT (missing baseline value).
- For energy intake data, a lot of data points were considered as not reliable because the mice played with the food and were excluded. In addition, data of 3 mice were excluded (H15, H22 and C14), and daily values below 5kcal or above 30kcal were also excluded. Data were analyzed between W4 and W12.
- For plasma free fatty acids, data from mouse Y12 were excluded from the analysis (missing baseline value).

Study 2:

- Two mice in Y group (Y7 and Y16) had to be euthanized at W13 due to their poor state following the OGTT. Data for these 2 mice were treated normally until W13.
- For energy intake data, a lot of data points were considered as not reliable because the mice played with the food and were excluded. In addition, data of 3 mice were excluded (Y11, C1, C23), and daily values below 5kcal or above 30kcal were also excluded. Data were analyzed between W4 and W14.

Study 3:

The following mouse values were excluded from data analysis:

- For energy intake, data points were considered not reliable if the mice played with their food and were excluded. Daily values below 5 kcal or above 30 kcal were also excluded. Data were analyzed between W4 and W11.
- C1 animal was excluded from the protocol on two criteria. First, it failed to gain body weight. Second, it was noticed during surgery that this animal had darker and viscous blood compared to the other animals. Based on these criteria, it was concluded that this animal was not representative of a "healthy" animal.
- The following mice were excluded from all clamp analysis due to death surgery-related before clamp or technical issues during HIE clamp procedure: C1, C2, C3, C5, C6, C8, C11, C15, C18, C19, C28, C29, C30, H3, H8, H11, H12, H16, H20, H21, H23, H32, Y3, Y4, Y5, Y12, Y15, Y18, Y20, Y24, Y27, Y28, Y32 and Y33.
- C21 mice: at the sampling time of 110 min, a stroke was induced in this animal (probably due to an air bubble injected in the carotid after the blood sampling). Therefore, all data points following this event cannot be used. However, the earlier time points are still relevant, and were kept in the analysis.
- The target euglycemia in the last 60 min of clamp was 7.0 ± 0.5 mmol/L. The mice C19, C28, H23 and Y12 were excluded because they didn't

reach euglycemia.
- A target insulin ratio of ≧ 2.0 ng/mL between basal and insulin stimulated (T-90 and T120 respectively) was adopted (posteriori to the HIE completion) to represent the clamp hyperinsulinemia. The mice C8, Y24 and Y32 were excluded for not reaching insulin ratio of ≧ 2.0 ng/mL.

Germ-free Fecal Material Transplantation Study:
- Results from one mouse in the H1-T group were removed from the analysis because of body weight loss due to male mice battle.
- Results from three mice in the H1-T-Chow group were removed from the analysis because of dramatic wounds following male mice battle.

Replication	To ensure the replication of our experimental findings we performed independent experiments, standardized and documented the experimental protocols, reduced and removed sources of bias.
Randomization	One week prior the study start, the animals were randomized for body weight into 3 treatment groups: C, H and Y.
Blinding	The investigators were blinded to group allocation for hepatic steatosis and fibrosis analysis to eliminate experimental biases. For the other parameter analyzed, the data collection and analyses wasn't blinded as the same persons conducted most animal care and subsequent analyses.

Reporting for specific materials, systems and methods

We require information from authors about some types of materials, experimental systems and methods used in many studies. Here, indicate whether each material, system or method listed is relevant to your study. If you are not sure if a list item applies to your research, read the appropriate section before selecting a response.

Materials & experimental systems

n/a	Involved in the study	
☒	☐	Antibodies
☐	☒	Eukaryotic cell lines
☒	☐	Palaeontology and archaeology
☐	☒	Animals and other organisms
☒	☐	Human research participants
☒	☐	Clinical data
☒	☐	Dual use research of concern

Methods

n/a	Involved in the study	
☒	☐	ChIP-seq
☒	☐	Flow cytometry
☒	☐	MRI-based neuroimaging

Eukaryotic cell lines

Policy information about cell lines

Cell line source(s)	L6 cells: gift from Amira Klip, Hospital for Sick Children, Toronto (Biochem J 1987 Feb15; 242(1); 131-136)<br>FAO cells: gift from Ronald Kahn, Harvard (Endocrinology 113:1201-1209, 1983)
Authentication	None of the cell lines used were authenticated.
Mycoplasma contamination	The cell lines were tested for mycoplasm contamination periodically.
Commonly misidentified lines (See ICLAC register)	No misidentified cell lines were used in this study.

Animals and other organisms

Policy information about studies involving animals; ARRIVE guidelines recommended for reporting animal research

Laboratory animals	Eight-week-old male C57Bl/6 mice.
Wild animals	The study did not involve wild animals.
Field-collected samples	The study did not involve field-collected samples.
Ethics oversight	All animal procedures were previously approved by the Laval University Animal Ethics Committee (Québec, Canada), referred to the 2016-004-21 protocol and followed the internal Guide for the care and use of laboratory animals.

Note that full information on the approval of the study protocol must also be provided in the manuscript.
